# Lack of α-synuclein increases amyloid plaque accumulation in a transgenic mouse model of Alzheimer's disease

**DOI:** 10.1186/1750-1326-2-6

**Published:** 2007-03-16

**Authors:** Verena Kallhoff, Erica Peethumnongsin, Hui Zheng

**Affiliations:** 1 Huffington Center on Aging, Baylor College of Medicine, Houston, TX 77030, USA; 2 Department of Molecular and Human Genetics, Baylor College of Medicine, Houston, TX 77030, USA; 3 Interdepartmental Program of Cellular and Molecular Biology, Baylor College of Medicine, Houston, TX 77030, USA

## Abstract

α-synuclein is a small soluble, cytosolic protein which associates with vesicular membranes. It is a component of intracellular Lewy bodies present in Parkinson's disease and a subset of Alzheimer's disease (AD). In addition, early studies identified a fragment of α-synuclein in the amyloid plaques of AD patients. Hypothesizing that α-synuclein might modify the AD pathogenic process, we crossed the Tg2576 strain of *APP *transgenic mice onto an α-synuclein knockout background to determine the effects of α-synuclein on Aβ production and plaque deposition. We found that α-synuclein deficiency does not affect the Aβ levels, nor does it alter the age of onset of plaque pathology. To our surprise, however, loss of α-synuclein leads to a significant increase in plaque load in all areas of the forebrain at 18 months of age. This is associated with an increase in another synaptic protein, synaptophysin. We thus conclude that α-synuclein is not involved in seeding of the plaques, but rather suppresses the progression of plaque pathology at advanced stages.

## Background

Alzheimer's disease (AD) and Parkinson's disease (PD) are the two most common age-dependent disorders of the central nervous system [1] conferring a detrimental neurodegenerative phenotype on the individual. In both diseases a subset of cases has been linked to autosomal dominant mutations in specific genes. While in inherited cases of AD, genetic mutations in amyloid precursor protein (*APP*) and presenilins (*PS1 *and *PS2*) lead to the pathological features of extracellular Aβ plaques and intracellular neurofibrillary tangles [2], mutations in the α-synuclein gene (*Snca*) confer familial PD, and its protein product is a component of the pathological hallmark, the intracellular Lewy bodies [3,4]. More than a decade ago evidence emerged that the Aβ plaques contain a peptide then termed "non-amyloid component of Aβ plaques" (NAC) [5,6], which was later identified as a fragment of α-synuclein. The 35 amino acid fragment found in the plaques was determined to be extremely hydrophobic [7], and multiple lines of evidence indicated that α-synuclein aids the aggregation of Aβ42 *in vitro *and might be involved in the formation of Aβ plaques in the brains of patients with AD and dementia with Lewy bodies (DLB) [8]. Studies by Yang et al. [9] suggested that α-synuclein was the major component accumulating in the neurites of aged Tg2576 *APP *transgenic mice and suggested that this aggregation might be caused by Aβ peptides. Other studies have generated *in vivo *data using overexpression of APP, leading to an increase of hydrophobic accumulations in a mouse model also overexpressing α-synuclein [10]. Together these published data indicate that α-synuclein might interact with other fibrillogenic proteins and enhance their accumulation and in return is positively influenced to aggregate as well. However, this detrimental function of α-synuclein has also been called into question as a few publications failed to establish any association between α-synuclein and Aβ pathology [11].

Although multiple α-synuclein transgenic lines have been created, which mimic some aspects of PD pathology [12], no studies have been reported on how the lack of α-synuclein affects the progression of neurodegenerative diseases. Several lines of α-synuclein (*Snca*) knockout mice have been established, most of which show only a very mild phenotype, suggesting genetic redundancy [13-15]. These studies suggested that α-synuclein is not an essential component of the neuronal system under normal circumstances or that other proteins may compensate for the loss of α-synuclein [16].

Based on published reports, we hypothesized that α-synuclein in an AD mouse model overexpressing *APP *would likely support the aggregation of Aβ plaques and its absence would result in a decreased number of plaques and a belated onset of pathology. We thus bred the *Snca *knockout mice [14] with Tg2576 [17] mice overexpressing mutant human APP that develop amyloid plaque pathology at 9–10 months of age, and the number of plaques increased dramatically with age [17].

## Results

**Figure 1:**
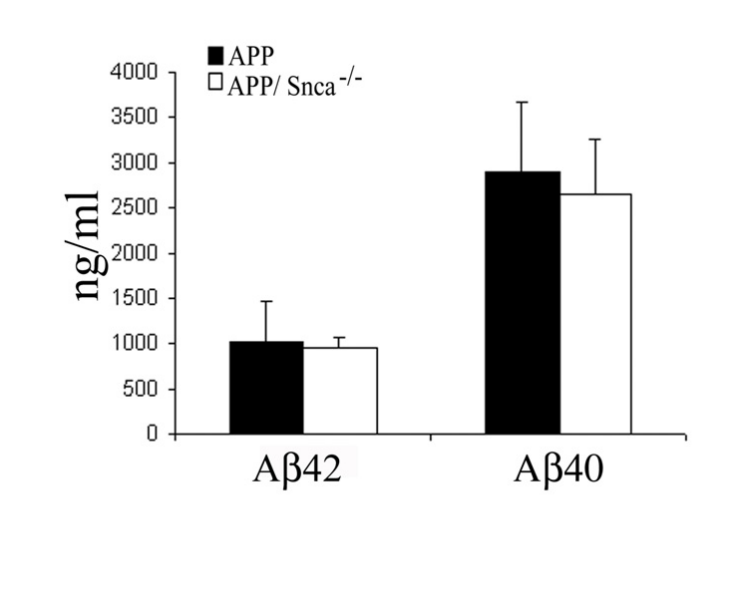
Sandwich ELISA of 6-month-old APP and APP/Snca^-/- ^animals. Absence of α-synuclein did not lead to significant alterations in the levels total Aβ40 and Aβ42.

In our study we used aged Tg2576 animals, herein referred to as APP animals, and compared their plaque pathology to that of APP animals lacking α-synuclein (APP/Snca^-/-^) at various ages. In general the mice are indistinguishable from their littermates in terms of appearance and viability. In order to determine whether we could detect differences in the production of two main Aβ species we performed Sandwich ELISA experiments at an early age. At 6 months of age neither the APP nor the APP/Snca^-/-^exhibit Aβ plaque pathology (data not shown). As expected, no difference in Aβ40 or Aβ42 levels could be detected by Sandwich ELISA (Figure 1) when comparing 6-month-old APP and APP/Snca^-/- ^animals. We then focused our study to determine the effects of α-synuclein on the onset of plaque deposition, and the progression of the pathology by comparing these parameters between APP and APP/Snca^-/-^.

**Figure 2:**
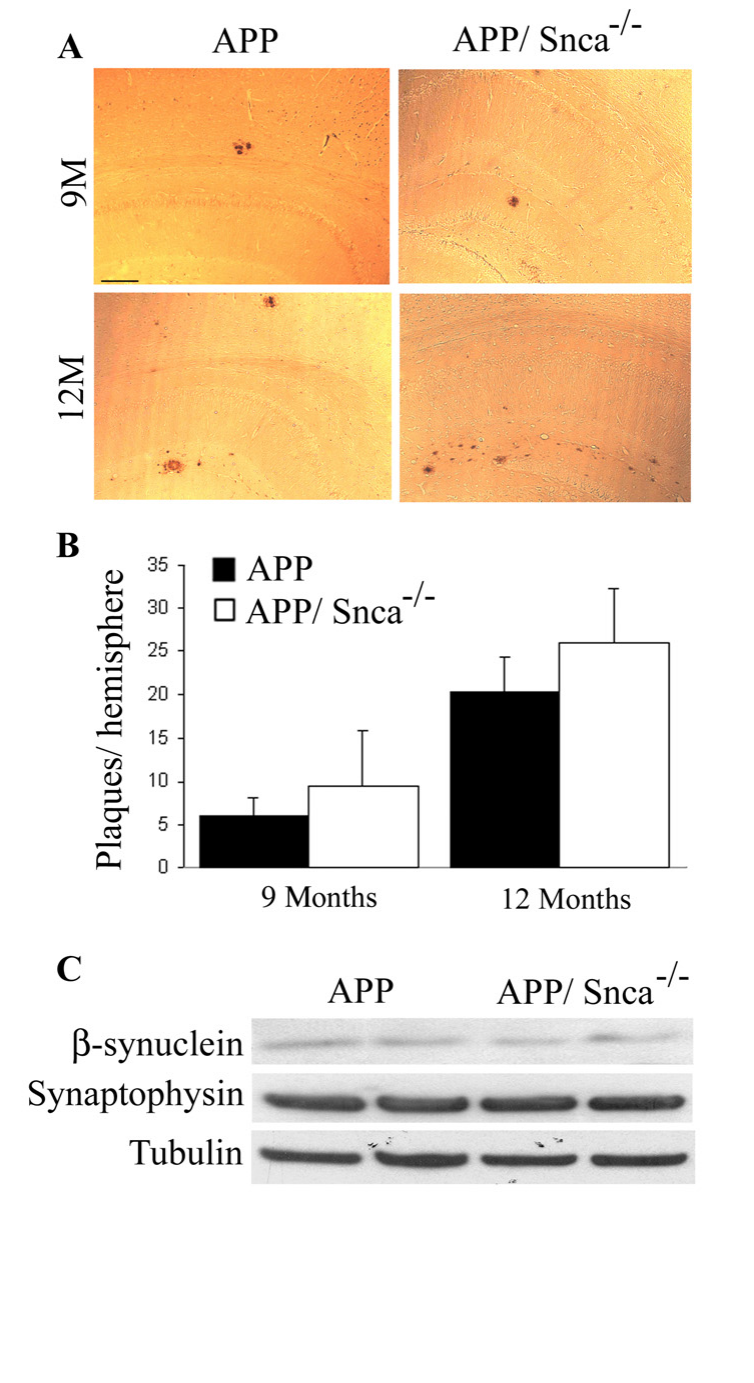
Immunostaining of 9- and 12-month-old APP and APP/Snca^-/- ^brains. A. Sections were stained with 6E10 antibody to detect the total number of amyloid beta plaques. Scale bar denotes 100 μm. B. Representation of the number of plaques per section per hemisphere. While the variability was much higher in the APP/Snca^-/- ^animals, there was no significant difference in the average number of plaques. N = 3 every 4^th ^section was examined. C. Western Blots of 12-month-old APP and APP/Snca^-/- ^brain homogenates. The protein was separated using 10% SDS-PAGE, transferred to a nitrocellulose membrane, and probed with antibodies against β-synuclein, synaptophysin and α-tubulin. No differences in protein expression could be detected.

In order to determine whether the age of onset of plaque deposition is affected, we studied 9-month-old APP and APP/Snca^-/- ^animals as that is the earliest time when plaques can be detected in APP mice. Using immunohistochemistry with 6E10 antibody to human APP, we detected a slight increase in the number of plaques (Figure 2A), but the difference failed to reach statistical significance (Figure 2B). The number of plaques increased in both APP and APP/Snca^-/- ^mice at 12 months (Figure 2). Similar to 9-month-old animals, there was a slight, but not statistically significant, difference between the two genotypes in the number or size of plaques (Figure 2B). As a homologous protein to α-synuclein, we studied the protein levels of β-synuclein as well as another synaptic vesicle protein, synaptophysin, in the brain homogenates of APP and APP/Snca^-/- ^mice. We were unable to detect significant differences in the expression of β-synuclein or synaptophysin in brain homogenates, limiting the possibility that these presynaptic proteins play a compensatory role for the loss of α-synuclein (Figure 2C). Overall, it appears that α-synuclein is not a large player in initiation/seeding of the plaque pathology in APP mice.

**Figure 3:**
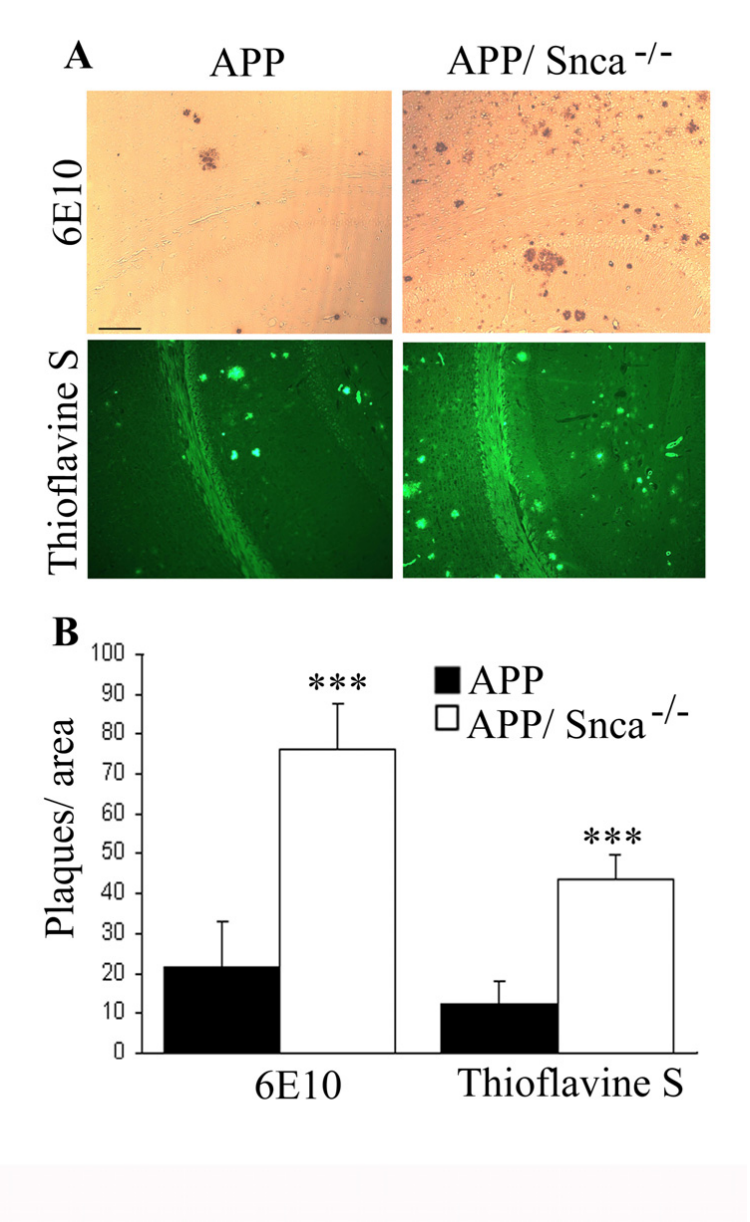
Plaque analysis of 18-month-old APP and APP/Snca^-/- ^animals. A. Sections were stained with 6E10 antibody or thioflavine S as indicated. 6E10 staining revealed the total plaque load while thioflavine S detected mature plaques only. The plaque density is much higher in mice lacking α-synuclein when compared to APP controls. Scale bar: 100 μm. B. Bar graph indicating the density of plaques per square area. The plaque density in the APP/Snca^-/- ^animals exceeds that of the APP animals by 3–4 fold. N = 4, ***p < 0.001

At 18 months of age, however, differences in the number of plaques became obvious. When performing 6E10 and thioflavine S staining of comparable coronal sections, we observed a vastly increased number of plaques in the APP/Snca^-/- ^animals when compared to APP animals (Figure 3A). Because the number of plaques in APP/Snca^-/- ^mice reached a level that was difficult to be counted manually, we chose to quantify the plaque density by assessing the signal intensity of matched areas. Statistical analysis revealed that both total (6E10-positive) and dense-core plaque (thioflavin S-positive) load in the APP/Snca^-/- ^animals were increased 3–4 fold when compared to the number of plaques found in age-matched Tg2576 animals (p < 0.001).

**Figure 4:**
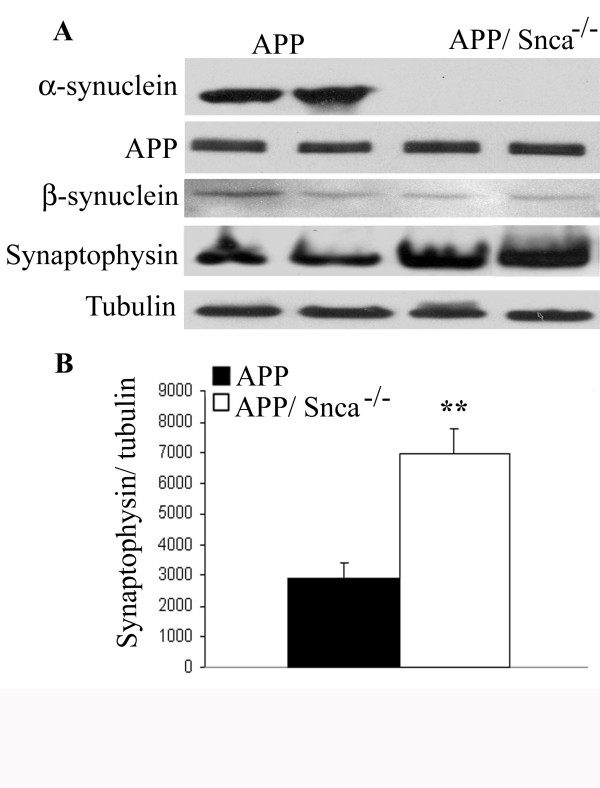
Western blotting of 18-month-old of APP and APP/Snca^-/- ^animals. A. 10 μg of forebrain lysates were separated on SDS-PAGE, transferred to a nitrocellulose membrane and probed with antibodies against α-synuclein, APP, synaptophysin and α-tubulin. No difference in APP or β-synuclein expression could be detected. α-synuclein was absent from APP/Snca^-/- ^animals, and synaptophysin levels were increase in APP/Snca^-/- ^animals. B. Quantification of band intensity of synaptophysin from APP vs APP/Snca^-/- ^animals. Data was normalized to tubulin bands (**p < 0.01).

As expected, Western blot analysis showed that α-synuclein protein was missing from the brain homogenates of the APP/Snca^-/- ^animals (Figure 4A) and the levels of full-length APP were not affected by the absence of α-synuclein. Once again we were unable to detect an increase in the β-synuclein (Figure 4A). Unexpectedly, we found that synaptophysin, which was unchanged at 12 months, was up-regulated by two-fold in APP/Snca^-/- ^animals when compared to APP animals (Figure 4B, p < 0.01). This could be attributed to a compensatory mechanism activated due to α-synuclein deficiency. However, the reason for the selective increase of synaptophysin, but not β-synuclein, is not clear.

## Discussion

Genetic mutations and gene amplification of α-synuclein (*Snca*) are linked to familial Parkinson's disease, and aggregation of the protein is the pathological hallmark of the disease. Numerous studies have focused on the effect that the overexpression of α-synuclein has on the dopaminergic system. In this pathogenic state, even as little as a two-fold increase in protein expression leads to the pathological feature of PD [12]. However, its normal function is much less understood. Early studies of knockout animals lacking either α- or β-synuclein or both revealed little impact on the mammalian system. Mice lacking the protein are phenotypically normal, and only close investigation reveals slight deficits such as a reduction in dopamine levels [12,13].

It is well established that α-synuclein localizes to the presynaptic terminals and associates with synaptic vesicles. In addition the protein has also been shown to preferentially associate with lipid rafts at these sites [18,19]. Lipid rafts are enriched in sphingolipids and cholesterol and are the site of localization for the γ-secretase complex [17], which is the enzyme responsible for the intramembranous cleavage of APP leading to the production of Aβ40 and Aβ42, among others [20]). Since α-synuclein localizes to the exact microdomain where APP processing takes place [17], it is a distinct possibility that the two proteins interact. Furthermore, research has suggested that α-synuclein expression is up-regulated in AD cases [21]. Overall these papers suggest that α-synuclein may be the primary insult leading to aggregation of the protein and subsequent neurodegeneration. While there exist several contradictory reports as to whether α-synuclein associates with Aβ containing plaques [9] it is likely that α-synuclein plays a contributing role in AD, PD and other diseases.

Over the years several publications have called into question the direct correlation between plaque and Lewy body pathology in AD and PD and the respective neuronal loss [22]). In addition, numerous papers recently suggested that a small increase in α-synuclein expression is the cellular response to oxidative stresses and confers a higher viability to the cells [23,24]. This sheds light on a different, neuroprotective property of α-synuclein in which the α-synuclein indeed is called upon in the times of cellular distress to help clear the insult [25]. In fact, our data that the amyloid plaques are increased in the absence of α-synuclein at old age suggests that α-synuclein may indeed serve as a chaperone helping the cells to clear protein deposits. While we were unable to detect any effect on the onset of plaque deposition in the APP animals, the distinct effect that α-synuclein has at a later stage of the pathology (18 months) is striking. Hence, α-synuclein does not appear to be involved in the onset of plaque deposition, but it is clearly involved in prevention of further accumulation in later stages of the pathology, maybe in clearing the Aβ fragments, possibly from the site of generation, to prevent further pathological development. Although many studies have suggested that α-synuclein is the culprit leading to neuronal cell death, it seems equally likely that under physiological conditions the protein presents a deterrent to the accumulation of other proteins whose accumulation might be more toxic to the cell. To date, α-synuclein has been shown to interact with a variety of proteins and is involved in a variety of diseases such as Parkinson's disease, AD, as well as Sandhoff disease [26], and it appears that the protein might play a more basic role in cellular defense from diseases. The role of the soluble protein might be to identify toxic proteins and bind to them to induce a cellular defense mechanism. In this scenario, under pathological conditions the amount of α-synuclein-containing aggregates is simply too much for the cellular machinery to handle, leading to intracellular aggregates as in the case of PD and Sandhoff disease or extracellular aggregates as in the case of AD. While many studies over the past year have indicated that α-synuclein has a protective role in neurons *in vitro*, to our knowledge this is the first report investigating the effect that the absence of α-synuclein has on the disease progression under pathological circumstances. There is no doubt that the aggregation of an otherwise neuroprotective protein could lead to pathological conditions if its natural response leads to protein aggregation and up-regulation of stress induced pathways. It is possible that the fact that α-synuclein was first identified as the protein whose overexpression leads to Parkinson's disease has masked its true role as a neuroprotector.

## Conclusion

Our study investigates the effect of the absence of α-synuclein in the progression of a pathological hallmark of AD. In this study we identified α-synuclein as a protein influencing the progression of plaque deposition in a transgenic mouse model of AD. Our data support a protective role of α-synuclein against amyloid pathology at a later stage, and offer an alternative explanation to the aggregation of α-synuclein under pathological conditions.

## Methods

### Mice

The Tg2576 *APP *transgenic mice and α-synuclein knockout mice have been described, and they were on B6SJL F1 and C57BL/6J backgrounds, respectively [14,17]. These mice were crossed to generate APP transgenic on α-synuclein heterozygous background, which were then crossed with α-synuclein heterozygous mice to produce APP transgenic mice on either wild-type or homozygous null for α-synuclein. The resulting animals were 87.5% C57 and 12.5% SJL. Animals were age- and sex-matched with a minimum N = 3 per study. All animal experiments were performed in accordance with the Baylor College of Medicine Institutional Animal Care and Use Committee and with national regulations and policies.

### Antibodies

The following antibodies were used in this study: to detect plaques and Aβ fragments 6E10 antibody (Signet, mouse monoclonal) was used. Synaptophysin (Dako Cytomation, rabbit polyclonal) antibody was used as a marker for pre-synaptic terminals, α-synuclein (BD Transduction Laboratories, mouse monoclonal) antibody was used to localize the protein of interest and ensure its absence in knockout animals, β-synuclein (Chemicon, rabbit polyclonal) and MAP2 (Sigma, mouse monoclonal) antibodies were used to locate post-synaptic termini; to ensure equal loading we used α-tubulin (mouse) antibody.

### Immunohistochemistry

All animals were anesthetized with avertin prior to perfusion and fixation in 4% PFA. Tissues were dehydrated using an ethanol gradient and subsequently paraffin embedded. Tissues were sectioned at a thickness of 6 μm. Wax was melted at 65°C and sections were deparaffinized in xylenes and re-hydrated via an ethanol gradient. Antigen retrieval was performed using citric acid treatment (1.8 mM citric acid, 8.2 mM sodium citrate) for 10 minutes boiling, and cooled at room temperature for 30 minutes thereafter. Sections were blocked with 10% Goat Serum and incubated with primary antibody overnight at 4°C. Secondary antibody incubation was performed at room temperature for one hour. To visualize plaques, 6E10 staining was developed using a horseradish peroxidase system (DAB, Vectastain); secondary antibodies for other stains were conjugated to a fluorophore. Images were taken using a Zeiss microscope and Axioskop software.

### Thioflavine S staining

Paraffin embedded sections were deparaffinized and rehydrated. Subsequently the sections were incubated in 0.01% thioflavine S in PBS for 10 minutes, decolorized with 2 washes of 95% ethanol and re-hydrated with PBS. Plaques were visualized using Axioskop (Zeiss) and Axiovision software. Plaque counts were performed manually (9 and 12 month old animals) or using Image J software (18 month old animals).

### Western Blotting

Brains were homogenized using NP40 lysis buffer (50 mM Tris pH 7.4, 150 mM NaCl, 10% Glycerol, 1% NP40) containing Complete Protease Inhibitor Cocktail (Roche). After 3 times 10 pulses of sonication the homogenates were spun at 14000 rpm for 1 hour. Protein concentrations were determined using Bio-Rad Dc Protein Assay (Bio-Rad). Equal amounts of protein were loaded on a 10% SDS-PAGE run at 100 V for 1.5 hours at room temperature and transferred onto a nitrocellulose membrane. Membranes were blocked one hour using 5% non-fat dry milk in PBS containing 0.1% Tween-20 (Sigma). Primary antibody incubation was done in blocking solution overnight at 4°C in constant agitation. Secondary antibody application was performed at room temperature for 1 hour. Bands were visualized using Immobilon™ Western ECL system (Millipore).

### Sandwich ELISA

Sandwich ELISAs for quantifying human Aβ40 and Aβ42 were performed as described using 6E10 as the capture antibody and AB112 and AB115 as detection antibodies which recognize Aβ40 and Aβ42 specifically [27]. Briefly, four volumes of TBS buffer (v/w) were used to homogenize brain samples, which were centrifuged at 8000 g for 1 hour. The pellet was resuspended in 5 M guanidine (2 volumes) and sonicated. 20 μl of homogenate was diluted with 10 volumes of loading buffer. After centrifugation at 8000 g for 30 minutes, the samples were loaded into wells for Aβ peptide detection. 100 μl of 6E10 (4 μg/ml) diluted in carbonate-bicarbonate buffer, pH 9.6, were added to coated microtiter plates and incubated at 4°C overnight. After washing plates with PBS containing 0.05% of Tween-20 (PBST), plates were blocked for 1 hour with 200 μl of PBST with 1% BSA. After washing the plate, 100 μl of standards was applied and incubated for 2 hours at room temperature or 4°C overnight. After washing, plates were incubated with biotinylated AB112 or AB115 diluted 1:1000 and 1:500, respectively, in PBST at room temperature for 1 hr 30 min. After washing, neutravidin-horseradish peroxidase (Pierce) diluted 1:5000 in PBST was added and incubated for 1 hour at room temperature. After washing the plate 3 times, 100 μl/ml of TMB solution was added and incubated for 15–30 min at room temperature. The color development was stopped by adding 100 μl/well stop solution (1 M phosphoric acid). The optical density (OD) was measured at 490 nm by a microELISA reader. The concentrations of Aβ40 and Aβ42 in the samples were calculated from the standard curve within the linear range for each plate, respectively. Six forebrain samples/genotype/age were analyzed each in triplicate.

## List of abbreviations used

AD: Alzheimer's disease; PD: Parkinson's disease; Aβ : β-amyloid; APP: amyloid precursor protein; PS: presenilin; DLB: Dementia with Lewy Bodies.

## Competing interests

The author(s) declare that they have no competing interests.

## Authors' contributions

VK generated the transgenic mice, carried out biochemical and immunohistochemical analyses, and wrote the first draft of the manuscript. EP assisted VK in the immunostaining, performed quantification of plaque load, and edited the manuscript. HZ is responsible in designing and overseeing the experiments and, together with VK, wrote the manuscript.
